# The prospect of tumor microenvironment-modulating therapeutical strategies

**DOI:** 10.3389/fonc.2022.1070243

**Published:** 2022-12-08

**Authors:** Dirk Eulberg, Anna Frömming, Kfir Lapid, Aram Mangasarian, Avital Barak

**Affiliations:** TME Pharma AG, Berlin, Germany

**Keywords:** tumor microenvironment, precision oncology, cancer immunology, cytokines, chemokines, angiogenesis, vasculogenesis

## Abstract

Multiple mechanisms promote tumor prosperity, which does not only depend on cell-autonomous, inherent abnormal characteristics of the malignant cells that facilitate rapid cell division and tumor expansion. The neoplastic tissue is embedded in a supportive and dynamic tumor microenvironment (TME) that nurtures and protects the malignant cells, maintaining and perpetuating malignant cell expansion. The TME consists of different elements, such as atypical vasculature, various innate and adaptive immune cells with immunosuppressive or pro-inflammatory properties, altered extracellular matrix (ECM), activated stromal cells, and a wide range of secreted/stroma-tethered bioactive molecules that contribute to malignancy, directly or indirectly. In this review, we describe the various TME components and provide examples of anti-cancer therapies and novel drugs under development that aim to target these components rather than the intrinsic processes within the malignant cells. Combinatory TME-modulating therapeutic strategies may be required to overcome the resistance to current treatment options and prevent tumor recurrence.

## Introduction

The classical definition of cancer spotlights fundamental cancer cell properties, including uncontrolled cell division, resistance to cell death, invasive and metastatic capacity, genomic instability and accumulation of cancer-driving mutations, and dysregulated metabolism and cellular signaling (1). Classically, drug development efforts have been invested in attenuating these characteristic cell-autonomous mechanisms with the hope of achieving clinically stable remission. Chemotherapeutic agents aim at halting mitosis and DNA synthesis or causing excessive DNA damage, leading to cancer cell death. The unfortunate aftermath is the collateral damage to healthy cells. To spare healthy cells, more advanced strategic concepts have emerged with the advent of targeted therapy. Cancer cells may elevate the expression of specific oncogenic proteins, making them attractive candidates for therapy (2). Well-known examples are (i) imatinib - a selective inhibitor of the abnormal BCR-ABL fusion kinase, characteristic of chronic myeloid leukemia (CML); (ii) bortezomib - a selective inhibitor of the 26S proteasome, vital for the survival of multiple myeloma (MM) cells due to their increased demand for protein turnover; and (iii) epidermal growth factor receptor (EGFR) inhibitors, such as erlotinib and gefitinib, approved for non-small cell lung cancer (NSCLC) (2). Another approach is cancer immunotherapy which revolutionized oncology by strategically harnessing the immune system to track and kill malignant cells. Exemplary strategies include (i) monoclonal antibodies (mAbs) directed at tumor-associated antigens and markers (e.g., human epidermal growth factor receptor 2 [HER2] and CD20); (ii) stimulation of effector leukocytes by means of immune checkpoint inhibitors (ICIs); and (iii) adoptive cell transfer of genetically engineered immune cells (e.g., chimeric antigen receptor T cells; CAR-T cells) (3, 4). Sadly, the cellular heterogeneity of tumors and their evolutionary nature can generally propagate resistance to different therapies (2–4).

Tumors are not semi-homogenous aggregates of cancer cells. In fact, in many tumors, malignant cells only represent a small minority of all cells. Tumors consist of various components, such as ECM, fibroblast-rich connective tissue, blood and lymphatic vessels, nerves, numerous subtypes of immune cells, and other types of mesenchymal cells. Collectively termed TME, the histopathological and molecular characteristics are utterly disparate from a normal tissue microenvironment. Thus, it does not come as a surprise that cancer cells are capable of utilizing and shaping the TME to their advantage (5). A thorough investigation of the molecular interactions between the tumor and its microenvironment, taking into consideration spatial and temporal cellular dynamics, may enable the development of novel therapies targeted at TME components rather than at the cancer cells. There is a need to apprehend how the TME of each anatomic cancer type differs from its equivalent normal tissue (either adjacent or from healthy controls). It is crucial to elucidate these pathophysiological differences because they play a role in tumor progression and resistance to therapy. Noteworthy, unlike publicly available cancer genome databases, no systematic studies have comprehensively compared the TME of various cancer patients. Notwithstanding, there is a general notion that the characteristics of each TME component are highly variable. This variability is not only due to differences between anatomic cancer types but also to intra-individual differences and intra-tumor heterogeneity.

Every tissue requires nutrients, oxygen, and waste clearance for survival and prosperity, which are naturally provided by the vascular system. These demands increase in hyperproliferative tissues, including tumors, and as a result, they turn hypoxic, triggering neo-vascularization. Tumors use two mechanisms to establish the necessary blood supply for the growing malignant tissue: angiogenesis and vasculogenesis. Angiogenesis is defined by the formation of new blood vessels from existing vessels. To promote angiogenesis, tumors are known to secrete pro-angiogenic factors, such as vascular endothelial growth factor (VEGF), fibroblast growth factor-2 (FGF2), and angiopoietins (6). This pathophysiological process is collectively termed the ‘angiogenic switch’ (7). In contrast, vasculogenesis is defined by the formation of new blood vessels following the recruitment and coalescence of bone marrow (BM)-derived, circulating endothelial progenitor cells (EPCs) (6). One of the key differences between malignant and normal tissues is the phenotypic appearance of a disorganized, leaky vasculature, owing to the rapid growth of capillary beds and their failure to mature fully (6). Disrupted endothelial integrity and enhanced permeability lead to uneven perfusion and interstitial pressure, which affects tumor physiology. In accordance, it also leads to reduced efficiency of drug delivery.

Cancer-associated fibroblasts (CAFs) are resident ECM-producing stromal cells that, by interacting with other TME components, acquire tumor-promoting properties, such as the secretion of growth factors and pro-angiogenic factors. Compared to normal fibroblasts, CAFs are considered to be strongly activated due to their enhanced proliferation capacity and secretory functions (8). This results in stromal rearrangements, such as desmoplasia, typified by the augmentation of myofibroblasts and dense, straightened ECM fibrils. Inflammatory fibrosis characterizes a significant portion of cancers, implying its contribution to carcinogenesis (9); however, fibrosis-low tumors are also highly influenced by CAFs. For example, CAF-derived matrix metalloproteinases (MMPs) degrade glycoprotein matrices, thereby enabling the release of bound growth factors and creating room for malignant cells to proliferate and migrate as well as vasculature to form (10). Moreover, CAFs may render the TME immunosuppressive by secreting transforming growth factor-beta (TGFβ), which represses lymphocyte and antigen-presenting cell (APC) effector functions (11). In doing so, CAFs remodel the local immune cell composition (8).

Teleologically speaking, malignant cells may maintain their abnormal self-identity and prosper if an anti-tumor immune response is avoided. Immune escape is signified by cell-autonomous mechanisms, such as loss of MHC class I expression or the elevated expression of inhibitory immune checkpoints (3, 4). Notably, immune escape can be the consequence of changes in the immune cell composition of the TME that affect both the adaptive and the innate immune systems. Escape from the adaptive immune system can be due to the inhibition of cytotoxic tumor-infiltrating lymphocyte (TIL) immunosurveillance and activation, enhanced by the enrichment of regulatory T (T_reg_) cells in the TME. T_reg_ cells are normally trained to recognize self-antigens, thus responsible for maintaining self-tolerance. However, since cancer cells commonly express self-antigens beside unique neoantigens, T_reg_ cells could, in principle, counteract the anti-cancer immune response (12). With respect to the innate immune system, the presence of myeloid-derived suppressor cells (MDSCs), tumor-associated macrophages (TAMs), and tumor-associated neutrophils (TANs) indicates an immunosuppressive TME associated with poor prognosis (13, 14). MDSCs and TANs originate from circulating BM-derived myeloid cells (13), while intra-tumoral TAMs either originate from tissue-resident macrophages or differentiate from recruited MDSCs/monocytes (14). All these cell types show similar immuno-modulatory functions, mediated by secreted factors [e.g., TGFβ and Interleukin-10 (IL-10)], metabolic reprogramming (e.g., arginine and tryptophan deprivation), or regulatory cell-to-cell contact (e.g., CD80/CD86) (12–14). Furthermore, TAMs and MDSCs support tumor vascularization and tumor progression by secreting pro-angiogenic factors and degrading the ECM, respectively (6, 13, 14). This highlights the complexity of the crosstalk between TME components that work in concert to maintain tumor viability.

**Figure 1:**
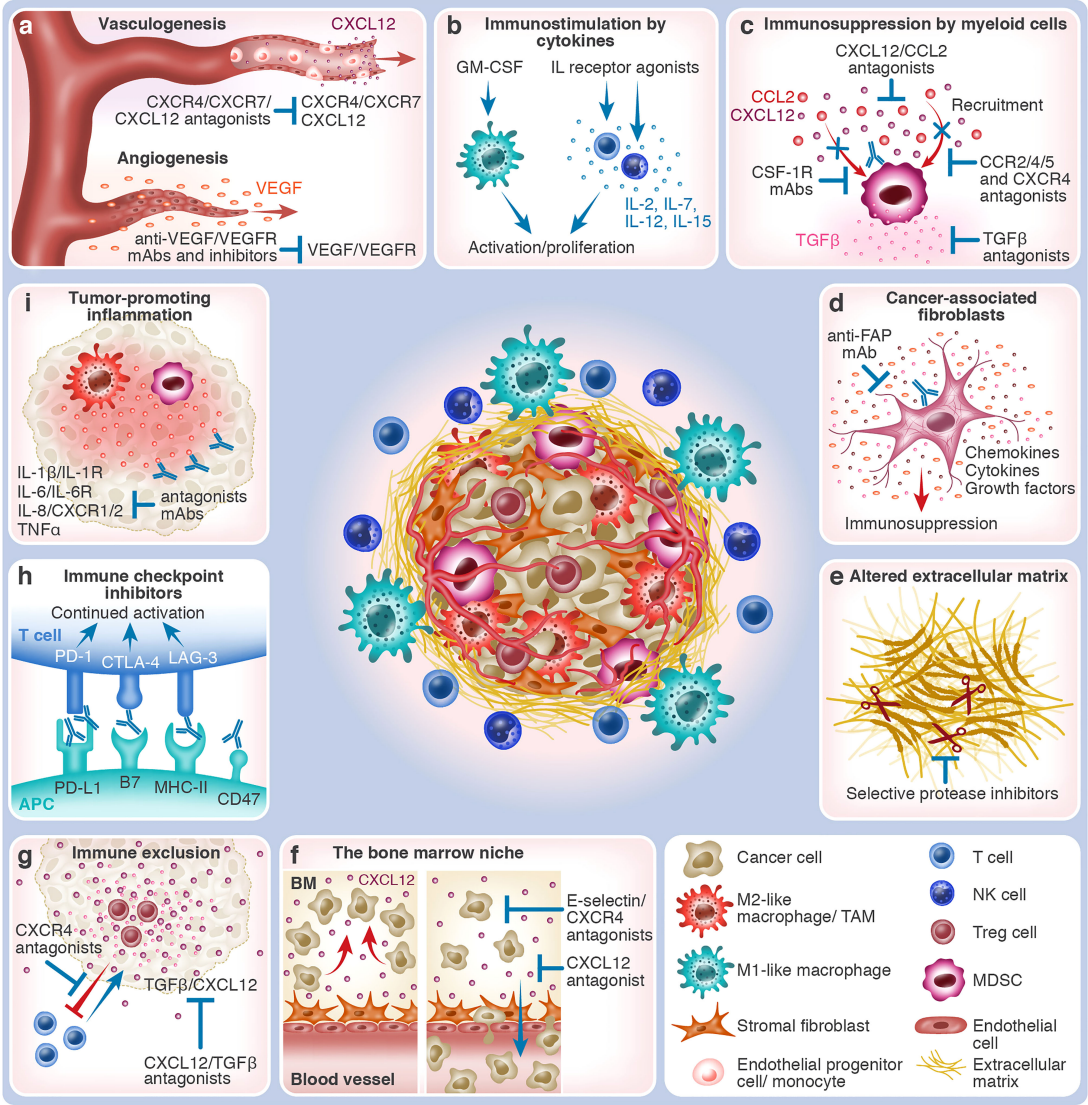
The various TME components and TME-targeting therapeutic strategies. The center of the figure depicts an immunosuppressed tumor which consists of malignant cells and different types of non-malignant cells that together constitute the TME. Immunosuppressive cells, such as MDSCs, M2-like macrophages, and T_reg_ cells, are shown within the tumor mass. At the periphery of the tumor mass are effector immune cells, such as T cells, NK cells, and M1-like macrophages. Blood vessels vascularize the tumor, which is also supported by stromal fibroblasts and altered ECM. The different tumor-promoting and anti-tumor TME processes and the respective interventional therapeutic strategies that target them are depicted in boxes around the tumor mass. Red arrows indicate tumor-promoting processes, whereas blue arrows & antibodies indicate therapeutic interventions. **(A)** Growth of blood vessels within the tumor in response to hypoxia. Vasculogenesis: a gradient of CXCL12 chemoattracts CXCR4^+^/CXCR7^+^ EPCs and BM-derived monocytes that contribute to the *de novo* growth of blood vessels and capillaries. CXCL12/CXCR4/CXCR7 antagonists (e.g., plerixafor and NOX-A12) block tumor vasculogenesis. Angiogenesis: VEGF is released, promoting vascularization from existing blood vessels and capillaries that nurture the tumor. Anti-VEGF/VEGFR mAbs and antagonists (e.g., bevacizumab and ramucirumab or TKIs) block tumor angiogenesis. **(B)** Immunostimulation by cytokines: In response to interleukins, such as IL-2, IL-7, IL-12, and IL-15, effector T-cells and NK cells undergo activation and proliferation, mounting anti-tumor immunity. The application of selective IL receptor agonists (e.g., ALT-803 and ALKS 4230), which have been developed to affect effector immune cells preferentially, stimulates this process. In response to growth factors, such as GM-CSF (e.g., produced by TVEC), M1-like macrophages and other APCs are recruited to the tumor site, assisting in the cross-activation of effector immune cells. **(C)** Immunosuppression by myeloid cells: TME-expressed chemokines, such as CCL2 and CXCL12, induce the recruitment of immunosuppressive myeloid cells, such as CCR2^+^/4^+^/5^+^ or CXCR4^+^ MDSCs and M2-like TAMs. Their immunosuppressive engagement is mediated by cytokines, such as TGFβ, that inhibit effector cell activity and, at the same time, activate suppressor cells. Therapeutic interventions can act upon several steps of this process: chemokine neutralization (e.g., NOX-E36 and NOX-A12) or blockage of their receptors by antagonists, elimination of myeloid cells (e.g., by targeting CSF-1R), or neutralization of TGFβ (e.g., SHR-1701). **(D)** Cancer-associated fibroblasts: CAFs and myofibroblasts are enriched in the TME, supporting tumor growth by secreting growth factors, altering the ECM, and creating an immunosuppressive microenvironment. Their elimination, for example, by anti-FAP mAb, has the potential to slow down or abrogate tumor progression. **(E)** Altered extracellular matrix: The TME is characterized by altered ECM properties, for example, the overexpression of the extra domain B of fibronectin (darkened segment). Often, tumors are associated with fibrosis and desmoplasia. Increased secretion of ECM-degrading proteases (illustrated as scissors), such as MMPs, heparanase, and uPA, enhances the invasion capacity of cancer cells. Targeting the altered ECM properties (e.g., L19 mAb) and the local administration of selective protease inhibitors could inhibit tumor expansion. **(F)** Protection of malignant cells in the bone marrow niche: Cancer cells of hematological origin utilize BM niches that, under normal conditions, support and protect HSCs. Retention in the BM is mediated by stroma-derived molecules such as CXCL12 or cancer cell-expressed adhesion molecules such as E-selectin. CXCL12/CXCR4 antagonists (e.g., NOX-A12 and motixafortide) and E-selectin inhibitors (e.g., uproleselan) cause loss of retention, which leads to the mobilization of cancer cells into the blood circulation, possibly promoting chemosensitization. Combining cell-killing agents with cell mobilizers could thus be more effective in eradicating hematological malignancies. **(G)** Immune exclusion: The effector CXCR4-expressing T cells are excluded from the tumor by CXCL12 gradients. The immunosuppressive TME, characterized by the presence, for example, of T_reg_ cells and TGFβ, also diverts effector immune cells, such as cytotoxic T-cells. CXCL12/CXCR4/TGFβ antagonists (e.g., plerixafor, NOX-A12, and bintrafusp alfa) may reverse tumoricidal effector cell exclusion by enabling their infiltration into the tumor mass. **(H)** Immune checkpoint inhibitors: The immune checkpoints CTLA-4 and PD-1, expressed by T cells, bind to CD80/CD86 and PD-L1/L2 on APCs or cancer cells, respectively, whereas LAG-3 on T cells binds to MHC-II molecules on APCs. Because immune checkpoints attenuate immune cell activation, ICIs (e.g., ipilimumab against CTLA-4, nivolumab/pembrolizumab against PD-1, atezolizumab against PD-L1, or relatlimab against LAG-3) instigate continued immune cell activation. The CD47/SIRPα pathway, employed by innate immune cells, hampers phagocytosis by APCs. Blocking this axis (e.g., by magrolimab or evorpacept) could thus enhance cancer cell recognition by the immune system. **(I)** Tumor-promoting inflammation: In some cancer entities, uncontrolled inflammation can cause effector cell exhaustion, thereby impeding effective anti-tumor immune response. Neutralization of pro-inflammatory cytokines, such as IL-1β, IL-6, IL-8, and TNFα, by mAbs, or blockage of their respective receptors, i.e., IL-1R, IL-6R, and CXCR1/2, facilitates resolution of tumor-promoting inflammation.

The comprehensive milieu of signaling messengers in the TME draws attention to the importance of creating a favorable microenvironment for cancer cells to thrive. In this review, we put emphasis on the CCL2/CCR2 and CXCL12/CXCR4/CXCR7 axes. On top of their supportive role in tumor growth and metastasis, they serve as important communication axes between TME components and malignant cells. The chemokines CCL2 and CXCL12 (previously known as monocytic chemotactic protein 1 (MCP-1) and stromal-derived factor-1 (SDF-1), respectively) are crucial for the attraction of CCR2^+^ and/or CXCR4^+^ MDSCs, T_reg_ cells and TANs as well as for the polarization of macrophages towards tumor-supportive TAMs (15, 16). Of interest, the chemokine CXCL12 has also been shown to exclude CXCR4^+^ effector T cells from the TME (16, 17). CXCL12 additionally promotes tumor vascularization by inducing endothelial cell differentiation, proliferation, and morphogenesis *via* its receptor CXCR7 (also called ACKR3) (16). Both CCL2 and CXCL12 are found to be highly secreted in a broad range of cancers and are often associated with poor prognosis, underscoring them as targets for anti-tumor therapy. We further explore the current strategies for targeting TME components as a conceptual therapeutical approach (**Figure 1**).

## Enhancement of anti-tumor immune response

Effector CD8^+^ T and NK cells routinely eliminate abnormal cells, which express tumor-associated antigens and tumor-specific neoantigens or have lost MHC class I expression. If a tumor is immunogenic (i.e., its malignant cells can be specifically recognized by the immune system), but the endogenous anti-tumor immune response is too weak or suppressed, a possible course of action is to enhance the immune response to achieve tumor eradication. Such a straightforward approach is the administration of immunostimulatory cytokines, cytokine receptor agonists, or the blockage of tumor-promoting pro-inflammatory cytokines (18). Given to patients already decades ago, interleukin-2 (IL-2), IL-12, IL-15, tumor necrosis factor-alpha (TNFα), and interferon-gamma (IFNγ) are potent immunostimulants of effector lymphocytes (18). However, the activity of cytokines is short-lived and highly pleiotropic, and their signaling pathways are often redundant, resulting in different responses in different cell types. Some of these responses are undesired and may lead to toxicity or even tumor-promoting effects. To address these limitations, biased receptor agonists have been developed. For example, ALKS 4230 (nemvaleukin alfa) is a fusion protein comprised of modified IL-2 and the high-affinity IL-2Rα chain. It preferentially triggers signaling *via* the IL-2Rβγ subunits, which activates cytotoxic CD8^+^ T and NK cells but not T_reg_ cells. In fact, the anti-tumor effect of ALKS 4230 in a mouse lung cancer model is superior to that of recombinant IL-2 (19). Displaying a similar selective immunostimulant mode of action, ALT-803 (N-803) is a ‘superagonist’ of IL-15R, a fusion protein of a mutant IL-15 and IL-15Rα-Fc. ALT-803 has been found to be effective in a mouse MM model (20) and other pre-clinical models. The adversity of cytokine-induced systemic toxicity, such as capillary leak syndrome, could be avoided by coupling the immunostimulatory cytokines to a targeted delivery system. The extra domain B of fibronectin is highly enriched in the ECM of many tumors, thus serving as a target candidate for cancer diagnosis and precision medicine, to which the specific antibody L19 has been developed (21). A series of novel biologics (e.g., darleukin, fibromun, and daromun) is based on the composition of L19 conjoined with recombinant IL-2 or TNFα. These well-tolerated compounds are currently being tested in several trials in combination with chemotherapy/radiotherapy for the treatment of metastatic melanoma, NSCLC, and soft tissue sarcoma (STS) (22, 23) (**Supplementary Table 1**). Another approach to elicit an enhanced immune response is to reduce immunosuppression, for instance, by neutralizing the immunosuppressive cytokine TGFβ (11). TGFβ is known to foster M2 polarization of TAMs and de-activate cytotoxic CD8^+^ T and NK cells. Selective inhibition of latent TGFβ1, the predominantly expressed TGFβ isoform in cancers, is efficient in potentiating the anti-cancer immune response in a murine model of refractory bladder cancer (24) but yet to be evaluated clinically.

A major mechanism by which an immunosuppressive TME is established is the upregulated expression of inhibitory checkpoints on malignant cells, CAFs, and immune cells (predominantly T cells, NK cells, macrophages, and dendritic cells (DCs)). These surface signaling molecules negatively regulate innate and adaptive immune cell responses. Therefore, substantial efforts have been invested in the development of biologics that inhibit these immune checkpoints, subsequently re-activating immune effector functions in the TME (3, 4). To date, the ICIs that have been approved for clinical use are mAbs that block programmed cell death protein-1 (PD-1), programmed death-ligand 1 (PD-L1), cytotoxic T-lymphocyte-associated protein-4 (CTLA-4), and lymphocyte activation gene-3 (LAG-3), all of which promote T-cell activity. The clinical development progress of ICIs that target other novel checkpoint molecules is discussed elsewhere (25). The co-inhibition of immune checkpoints may add to efficacy, for example, the anti-PD-1 nivolumab plus the anti-CTLA-4 ipilimumab in the treatment of NSCLC (26). Of note, this combination of ICIs has a less favorable safety profile than either of the mAbs alone. Unfortunately, a large fraction of cancer patients is unresponsive to ICIs due to (i) a low expression of immune checkpoint receptors/ligands; (ii) a low inflammatory profile of the TME; or (iii) T-cell exhaustion, characterized by poor effector functions (3, 4, 27). Another attractive yet-to-be-approved therapeutic strategy targets ‘myeloid checkpoints’. For example, the CD47/signal-regulatory protein α (SIRPα) pathway transduces a ‘don’t eat me’ signal to inhibit phagocytosis by SIRPα-expressing macrophages/DCs. Thus, the surface CD47 expression by cancer cells plays to their advantage by avoiding phagocytes, cancer-associated antigen presentation, and, eventually, T-cell priming (28). The leukocyte immunoglobulin-like receptor subfamily B (LILRB) comprises immunomodulatory receptors that recognize the ubiquitously expressed MHC class I molecules, which are responsible for distinguishing self from foreign (29). Anti-CD47/SIRPα/LILRB mAbs or antagonists as modalities to elicit tumoricidal immune responses have shown success in animal models and are under clinical investigation (28, 29). Therefore, the assessment of innovative combination therapies applying ICIs, also against innate checkpoints, is warranted (**Supplementary Table 1**). An example of such an innovative approach is the design of HX009, a bifunctional anti-CD47/PD-1 mAb, currently assessed in early studies (30).

Cancer cells are capable of developing immune evasion mechanisms; therefore, the tumor adapts to acquire ‘cold’ properties (i.e., lack of effector immune cells in the tumor parenchyma) (3, 4). ‘Cold’ tumors are categorized into two types: (i) ‘immune–excluded’ tumors, where effector TILs accumulate in the tumor periphery but are unable to infiltrate due to the immunosuppressive TME; (ii) ‘immune-desert’ tumors, where also the surrounding tissues are devoid of effector TILs due to low immunogenicity. In these cases, the non-targeted approach of immunostimulants as monotherapy is likely to be therapeutically futile. Combination therapy of immunostimulants together with ICIs could be promising in paving the way to overcome localized immunosuppression entailed by the TME. In particular, immune-excluded tumors could be reverted to ‘hot’ tumors (i.e., inflamed with a high presence of TILs). When administered together with ICIs, these aforementioned immunostimulant drugs have shown a degree of success not only in pre-clinical cancer models (31–33) but also in clinical trials (**Supplementary Table 1**). Following observations of robust TIL recruitment and benefits of overall response in earlier studies, ICIs like nivolumab and pembrolizumab (anti-PD-1 mAbs) are currently being tested in conjunction with ALKS 4230 (34), ALT-803 (35), or other immunostimulants, in phase 2/3 trials of patients suffering from advanced cancers (**Supplementary Table 1**). Ineffective alone, TGFβ antagonists could also be combined with ICIs to allow cytotoxic T cell penetration to the tumor core, consequently mounting anti-tumor immunity (33). Bintrafusp alfa (M7824) merges these two therapeutic strategies into one dual-purpose drug: a bifunctional fusion protein composed of the extracellular domain of TGFβRII, which can trap free ligands, and an anti-PD-L1 mAb (36). It is now clinically tested for several oncological indications (**Supplementary Table 1**). Taken together, these therapeutic innovations highlight the importance of the immune cell composition of the TME in the accomplishment of anti-tumor effects, as discussed next.

## Modulating the immune TME

Altering the immune cell composition of the TME could be a fruitful strategy to prevent tumor progression if these interventions materialize: (i) the selective depletion of immunosuppressive cells, such as T_reg_ cells, M2-poised TAMs, TANs, or MDSCs, from the TME; (ii) the alleviation or resolution of the intense, non-specific TME inflammation, clinically evident in some malignancies; (iii) the increased trafficking of effector T and NK lymphocytes or APCs to the tumor site. Effector TILs may be trapped in the tumor stroma (immune-excluded tumors) or may not be present (immune-desert tumors) (3). Thus, to develop effective immune TME-targeting strategies, scientists investigate the eminent functions of signals mediated by growth factors, cytokines, and chemokines affecting immune cell trafficking. The comparative scrutinization of receptor-ligand repertoires and their downstream effects in different immune cells, whether pro- or anti-tumor, is one of the main principles guiding oncological drug development today.

A straightforward approach is to eliminate immunosuppressive cells. The elimination of T_reg_ cells by targeting highly expressed markers, such as CCR4 or CD25 (i.e., the IL-2Rα chain), is under investigation (12, 18). So far, however, more efforts have been directed toward the elimination of immunosuppressive myeloid cells. Colony-stimulating factor-1 (CSF-1; also known as macrophage-CSF, M-CSF) drives monocyte differentiation and proliferation and supports macrophage survival (14). Although not specific to TAMs and MDSCs, the overall effect of blocking its receptor CSF-1R is potentially beneficial. Anti-CSF-1R mAbs and CSF-1R inhibitors have been shown to reduce CD68^+^/CD163^+^ TAM numbers in solid tumors pre-clinically as well clinically (37–39). While in most pre-clinical experiments, the anti-tumor effect of CSF-1R inhibitors is compelling, no or only marginal objective clinical responses have been demonstrated in early clinical trials (as monotherapy or with chemotherapy) (14). This might be attributed to the incompetence to modify immune cell composition in the TME in a way that favors anti-tumoral immune cells over pro-tumoral immune cells (40, 41). A phase 2 trial in advanced pancreatic cancer patients, treated with the anti-CSF-1R mAb cabiralizumab and the ICI nivolumab, has not met its efficacy endpoint (42). This result, however, does not exclude success in other ongoing trials (**Supplementary Table 1**). As mentioned above, CCL2 is a key chemoattractant of CCR2^+^ monocytes that can differentiate into tumor-supportive MDSCs and TAMs (15). In a similar manner to CSF-1R blockage, anti-CCR2 mAbs and CCR2 antagonists prevent TAM and MDSC infiltration, accompanied by elevated TILs, in animals and humans alike (43–45). Current clinical trial results in treating advanced unresectable pancreatic cancer are promising (45–47), warranting further investigation. Of interest, blocking the ligand CCL2 itself might be an alternative approach (see **Box 1**).

Since the CCL2/CCR2 axis is pivotal in inflammatory processes, it is worth evaluating how intervention by modulating the inflammatory properties of the TME can augment anti-tumor immunity. In many cancer entities, chronic inflammation and lymphocyte exhaustion are considered to be feed-forward reactions to the tumor burden and ineffective anti-tumor immunity (48). This allows the neoplasm to maintain its pathology. The underlying pathophysiological mechanisms include tissue damage and mutagenesis, ECM breakdown and fibrosis, the release of mediators that support cancer cell growth and invasiveness, and myeloid cell-mediated immunosuppression of effector lymphocytes (48). Attempts have been made to block pro-inflammatory cytokines, primarily IL-1β, IL-6, IL-8, and TNFα, or their receptors using mAbs or specific antagonists. These attempts have mostly concluded in no or only modest clinical success (18). The anti-IL-1β mAb canakinumab remarkably lowers lung cancer mortality (49), but its unfavorable immunocompromising effect in patients results in increased exposure to hazardous infections (50). Recent advances have been made by administering combinatory regimens in animal models (51–55) and relapsing patients with drug/immunotherapy-resistant tumors (**Supplementary Table 1**). It is apparent that the TNFα inhibitors infliximab and etanercept, given as prophylaxis or salvage treatments, potentiate susceptibility to ICIs. More importantly, they reduce severe adverse effects related to ICI-induced autoimmune inflammation (56, 57), e.g., colitis in melanoma patients and pneumonitis in lung cancer patients (58). Thus, targeting the TME can not only support an effective anti-tumor immune response but also alleviate treatment-related comorbidities. Depending on the tumor type and TME characteristics, inhibition of inflammation could be either productive or unproductive, further discussed elsewhere (3, 48).

An innovation leap has been made when the notion of adoptive cellular therapy was introduced. Basically, tumor-reactive immune cells, such as T cells and NK cells, are cultured, expanded, exogenously activated, or genetically engineered, followed by re-infusion into the patient. The most advanced approach is CAR-T cell therapy, in which T cells are armed with a chimeric receptor composed of a mAb-derived antigen-binding domain designated for a specific tumor-associated antigen and intracellular TCR-derived signaling and costimulatory domains (59). At present, the medical indications are mainly hematological malignancies. In solid tumors, the obstacles are both the selective pressure to lose surface expression of the targeted antigen and the limited infiltration of transferred CAR-T cells into the tumor tissue. To overcome this obstacle in immune-excluded tumors, scientists plan to deliver CAR-T cells together with ICIs or design CAR-T cells that also secrete immunostimulatory cytokines (59). Notably, the current development of M1-like CAR-macrophages for clinical use has the potential to compete with immunosuppressive M2-like TAMs and MDSCs in the TME (60). Furthermore, administering medicinal agents to overcome immune cell exclusion could presumably enhance the efficacy of immunotherapies, including adoptive cell therapy.

Provoking recruitment of endogenous effector cells to the tumor is an alternative to adoptive cell transfer, attaining a similar anti-tumor immune response. This can be achieved following the administration of a cytokine, a chemokine, or a growth factor that essentially supports effector leukocytes. A unique drug delivery technique employs oncolytic viruses to express drug proteins within the TME. The FDA/EMA-approved talimogene laherparepvec (TVEC), which utilizes oncolytic herpes simplex virus (oHSV) as a vector, has been developed to treat melanoma through direct injection into the tumor lesion (61). The genetically engineered oHSV, which preferentially replicates in cancer cells, also encodes the human granulocyte-macrophage colony-stimulating factor (GM-CSF). GM-CSF is released from bursting infected cancer cells; in turn, it entices professional APCs which ultimately activate anti-tumor T cells. The concept of oHSV-mediated immunovirotherapy is reviewed in (61) (also see **Supplementary Table 1**: ongoing clinical trials of TVEC + ICIs). One may even speculate whether a particular TME-expressed signaling molecule is capable of repelling TILs; therefore, blocking its signals would potentially lead to an influx of effector TILs. The CXCL12/CXCR4 axis has been identified to play roles in both TIL chemorepulsion and MDSC chemoattraction, placing it as a dual-purpose target for TME therapy (16, 17). In animal models, selective CXCR4 inhibition has been extensively investigated in combination with other pharmaceuticals, demonstrating therapeutic feasibility (16). In humans, the CXCR4 antagonist motixafortide (BL-8040) plus pembrolizumab have shown clinical efficacy in a phase 2 trial of chemotherapy-resistant pancreatic ductal adenocarcinoma (PDAC) (62, 63). T-cell-inflamed TME was displayed as a treatment response. Despite encouraging early results in relapsing HER2-negative metastatic breast cancer patients (64), the CXCR4 antagonist balixafortide plus the chemotherapeutic macrocycle eribulin have not shown any added therapeutic value in a phase 3 clinical trial. Interestingly, blocking the ligand CXCL12 itself, which inhibits the interaction of CXCL12 with both of its receptors, CXCR4 and CXCR7, might be a pharmacologically superior approach to targeting this axis (see **Box 1**). The therapeutic approach of utilizing CXCR4 inhibition to combat hematological malignancies is separately discussed under the section “Targeting the niche”.

## Angiogenesis and vasculogenesis inhibitors

Recognizing that the persistence of highly vascularized tumors depends on the ‘angiogenic switch’ has prompted the development of angiogenesis blockers with the therapeutic goal of impoverishing tumor blood supply (6, 7). The anti-VEGF-A mAb bevacizumab was the first angiogenesis blocker approved as a complementary anti-cancer therapy. It was followed by other biologics that target the VEGF/VEGF receptor (VEGFR) axis as well as a series of small-molecule receptor tyrosine kinase (RTK) inhibitors (TKIs). TKIs can inhibit angiogenesis in addition to direct effects on malignant cells (e.g., inhibition of cell proliferation and induction of apoptosis) (65). The RTK superfamily encompasses dozens of growth hormone receptors: (i) VEGFRs, FGF receptors (FGFRs), and mesenchymal-epithelial transition factor (c-Met) that promote endothelial cell proliferation and migration; (ii) c-Kit that marks endothelial progenitor cells; and (iii) platelet-derived growth factor receptors (PDGFRs) that support blood vessel-conjoined pericytes and smooth muscle cells. As a mode of action, TKIs multi-target RTKs by competing with ATP over the conserved ATP-binding domain (65). Therefore, TKIs are regarded as non-specific despite some observed variable selectivity. A third therapeutic approach targets endogenous anti-angiogenic mechanisms that regulate endothelial cell proliferation, migration, and survival (6). Pharmaceutical recombinant proteins, peptides, and gene therapy techniques have been developed based on various endogenous anti-angiogenic factors, such as endostatin, angiostatin, and thrombospondin [reviewed in (66–68)]. To date, treatments targeted toward endogenous anti-angiogenic factors have not been approved as cancer therapy modalities in the western world.

In spite of the established relevance of angiogenesis for tumor growth, anti-angiogenesis medications largely demonstrate limited effects on overall survival as a result of intrinsic or acquired drug resistance. Moreover, in congruence with experimental models, tumor vascularization has been shown to re-commence upon anti-angiogenesis drug withdrawal, leading to increased malignant aggressiveness (6). A therapeutic strategy to confront anti-angiogenesis resistance is the parallel or successive administration of several anti-angiogenesis drugs; it is yet unknown if this approach results in a clinical benefit. A double blow to halt cancer progression may be achieved by combining anti-angiogenesis therapy with drugs that target other TME components. In unresectable hepatocellular carcinoma (HCC) and metastatic NSCLC patients, a first-line combined therapy of chemotherapy, bevacizumab, and the anti-PD-L1 mAb atezolizumab is superior to that of chemotherapy plus anti-angiogenesis treatment alone (69, 70) (more examples in **Supplementary Table 1**). Favorable clinical outcomes are also observed in metastatic renal cell carcinoma (RCC) patients treated with ICIs and TKIs compared to those treated with either medication alone (71–73).

Diverse mechanisms can drive tumor vascularization. Alternative non-angiogenic courses of tumor vascularization, such as vascular mimicry (i.e., tumor cells that form vessel-like structures), co-option of existing blood vessels, or *de novo* vasculogenesis by EPCs, could take over (6). As a result, continued blood supply is enabled despite conventional angiogenesis blockade. Thus, implementing targeted inhibition of several pathophysiological processes of tumor vascularization could yield better clinical outcomes than targeting angiogenesis alone. As a result of high energy demands, nutrient deficits, and irregular blood supply, TMEs are typically characterized by hypoxia (6). Hypoxia, *via* stabilization of the transcription factor hypoxia-inducible factor-1 alpha (HIF-1α), upregulates the expression of pro-angiogenic/vasculogenic factors, such as the chemokine CXCL12 (16). In turn, CXCL12 presentation in the TME and its resulting concentration gradients attract circulating CXCR4/CXCR7^+^ EPCs and promote tumor re-vascularization. This is especially noted under the pathological settings of hypoxia/inflammation-induced CXCR4 and CXCR7 upregulation. Significantly, the CXCL12/CXCR4/CXCR7 axis functionally contributes to cancer cell motility, invasiveness, and metastasis (16). Thus, the inhibition of this axis may be advantageous by targeting both tumor vasculogenesis and the cancer cells directly.

Strikingly, VEGF/VEGFR inhibition elevates CXCL12 and CXCR4 expression in the TME of colorectal cancer (CRC) and glioblastoma (74, 75), thus bypassing VEGF-dependent angiogenesis blockade and contributing to tumor recurrence. In experimental murine models, co-treatment with CXCR4 and VEGF inhibitors overcomes this bypass, exerting a synergistic effect on limiting tumor growth (75–78). The observation that CXCR7 inhibition alone demonstrates an anti-vasculogenic effect *in vivo* suggests that it may be beneficial to block the whole CXCL12/CXCR4/CXCR7 axis to achieve full efficacy (79–81). However, research is still needed to examine whether CXCR7 action is associated with the observed resistance to VEGF/VEGFR blockade therapy. Among multiple CXCR4 inhibitors available for research, only AMD3100 (also known as plerixafor) has been approved for the induction of autologous hematopoietic stem cell (HSC) mobilization in patients with hematological malignancies (see ‘targeting the niche’). Given the established role of the HIF-1/CXCL12/CXCR4 pathway in tumor vasculogenesis, antagonists to this pathway are predicted to display clinical potency as anti-vasculogenic agents. Indeed, plerixafor infusion or an administration of an anti-CXCR4 mAb have shown the capability of inhibiting post-irradiation tumor revascularization in mouse and human glioblastoma (82, 83). To date, the vast majority of oncology studies evaluate the clinical efficacy of CXCR4 antagonists based on their prominent anti-tumor or mobilizing effects (16) and not anti-vasculogenesis effects. Of interest, neutralizing the CXCL12 ligand itself might be an alternative approach that allows signaling blockade through both CXCR4 and CXCR7 (see **Box 1**).

## Targeting the structural elements of the surrounding stroma

A pathological tissue repair process may commence in the cascade of events resulting from hypoxia and cellular damage, in particular following cancer chemoradiotherapy. In response to the partially damaged malignant tissue, the stroma is re-organized by various supporting mesenchymal cells. CAFs and myofibroblasts, abundant in almost all epithelial cancers, are as important as endothelial cells in promoting tumorigenesis and post-chemoradiotherapy tumor recurrence (5, 8). A targetable candidate CAF marker is fibroblast activation protein (FAP), a membrane-bound protease that functions in tissue repair by degrading ECM glycoproteins. FAP is enriched in CAFs but lowly expressed in the stroma under homeostatic conditions (84). As with other standalone TME targets, anti-FAP therapy does not appear to be very promising as a monotherapy. In autochthonous models of highly fibrotic PDAC, the tumors are unresponsive to ICIs unless pre-conditionally depleted of FAP^+^ CAFs (85, 86). The underlying mechanism involves CAF-expressed CXCL12-mediated immunosuppression, shown to be offset by the CXCR4 antagonist AMD3100 (86). This is corroborated by the finding that the CXCL12/CXCR4 pathway mediates CAF-dependent immunosuppression in a fibrotic breast cancer model (87). In that study, AMD3100 diminishes desmoplasia and enables effective immunotherapy in otherwise resistant tumors (87). Simlukafusp alfa (FAP-IL2v, RO6874281) is a chimeric biologic composed of anti-FAP mAb and an IL-2 variant with a selective affinity for IL-2Rβγ. It demonstrates synergistic efficacy in tumor-bearing animals when combined with various immunotherapeutic drugs (88). Clinical outcomes from early clinical trials lie ahead.

FAP being an ECM-degrading protease raises the question of whether inhibiting other stromal proteases could serve as a therapeutic strategy. Not only CAFs but also cancer cells, TAMs, and other TME-residing cells produce ECM-degrading proteases, including MMPs, urokinase plasminogen activator (uPA), and heparinase; all of which are associated with poor prognosis (10, 14). Released ECM-bound growth factors and bioactive ECM fragments can, in turn, initiate tumor-promoting/metastatic biological processes. The usefulness of non-selective MMP inhibitors is limited by their tendency to cause musculoskeletal and gastrointestinal derangements and have so far failed to show any objective clinical response (89). Conversely, selective MMP inhibitors with improved pharmacokinetics and toxicity profiles have translational potential for metastatic cancer (89); confirmation is still needed in clinical studies. In a phase 3 clinical trial conducted in gastric and gastroesophageal cancer patients, the addition of anti-MMP-9 mAb andecaliximab to standard chemotherapy has not been proven to increase efficacy (90). A similar discouraging clinical outcome was observed when andecaliximab was combined with nivolumab (91). Success may depend on the careful selection of a combination treatment protocol according to the patient’s diagnosis/prognosis profile. Roneparstat, a potent heparanase inhibitor, has shown the potential to overcome chemotherapy resistance in human MM cells (92). So far, uPA inhibitors have shown low selectivity and potency; however, a highly selective novel analog capable of eradicating metastases in a xenograft model of PDAC (93) could be of translational value. In conclusion, the key mechanistic message here is that protease inhibitors could be useful in coping with metastatic tumors, muting invasive properties, rather than coping with tumor growth directly.

## Targeting the niche

The BM contains microanatomical sites, termed stromal niches, that support vigorous hematopoietic cell proliferation and differentiation under homeostasis or upon demand. These supportive niches grant protection from oxidative stress and other insults and are tightly regulated (94). Remarkably, malignant cells, especially those of a hematological origin, exploit and remodel stromal niches, through which they acquire abilities of improved cell survival, immune evasion, and resistance to chemoradiotherapy. Theoretically, the active detachment and displacement of malignant cells from stromal niches into the blood circulation can increase cellular vulnerability to the cytotoxic effects of any anti-cancer therapy. Malignant cells employ similar migration, adhesion, and homing mechanisms into the BM as normal hematopoietic cells, for example, the involvement of the CXCL12/CXCR4 pathway in mediating HSC retention in the BM. Thus, the therapeutic strategy is to induce mobilization of malignant cells into the peripheral blood (PB) in the same manner as practically done for HSCs in preparation for apheresis. Mobilization can then be followed by radiotherapy, chemotherapy, targeted therapy, or immunotherapy with the goal of eradicating the now unprotected malignant cells. Granulocyte colony-stimulating factor (G-CSF) and the CXCR4 antagonist plerixafor (in conjunction or alone) are well-established HSC mobilizing agents for both allogeneic and autologous transplantations. Likewise, these agents display the capacity to mobilize CXCR4^+^ human leukemic and MM cells to the PB (94, 95). The application of this mobilization/chemosensitization strategy has been tested in patients suffering from relapsed/refractory (R/R) acute myeloid leukemia (AML) and MM, in whom complementary chemotherapy has shown favorable results (96–98) (see also **Supplementary Table 1**). Furthermore, scientists have evaluated the direct immunotherapeutic impact of the anti-CD20 mAb rituximab on human chronic lymphocytic leukemia (CLL) cells in response to the co-administration of CXCL12/CXCR4 inhibitors (99, 100). Mechanistically, it is unclear whether mobilizing malignant cells into the PB per se is sufficient to increase their chemosensitivity. In laboratory research, G-CSF and CXCR4 antagonists can directly stimulate cell cycle entry and susceptibility to apoptosis in hematological malignant cells, suggesting another plausible cellular mechanism to explain the observed augmented chemosensitivity (95). Notably, not all malignant blasts express CXCR4 on their surface, making this therapeutic strategy occasionally ineffective. Yet, given the findings that CXCR4 expression often correlates with malignancy grade, plerixafor-responsive cancer patients presumably would have had otherwise unfavorable prognoses. While most pharmacological approaches have relied on CXCR4 blockade, inhibiting the ligand CXCL12, which also blocks interaction with CXCR7, is an alternative approach for mobilizing malignant cells (see **Box 1**).

Various adhesion mechanisms enable retention in stromal niches. Therefore, impeding these adhesion interactions is instrumental in achieving loss of retention and peripheral mobilization of adherent cells, whether normal or malignant. The interruption of anchorage-dependent cell signals can, as a result, induce apoptosis in malignant cells that depend on their protective niches for survival (i.e., anoikis or ‘death by neglect’) (94, 101). One of these mechanisms is mediated by the vascular adhesion molecule endothelial selectin (E-selectin; CD62E). Expressed in the BM microvasculature, E-selectin binds to sialylated ligands on HSCs as well as leukemic cells, promoting chemoresistance of the latter (102). When combined with chemotherapy, the E-selectin antagonist uproleselan (GMI-1271) leads to high remission rates in patients with R/R AML (103). This approach is currently being tested in a randomized phase 3 trial (**Supplementary Table 1**). Based on preclinical experiments, uproleselan is predicted to also potentiate mobilization/chemosensitivity in chronic myelogenous leukemia (CML) and MM (102). Interestingly, a novel dual CXCR4/E-selectin antagonist (GMI-1359) is a powerful mobilizing agent with therapeutic potential (104, 105). At the infancy of clinical development for the treatment of hematological malignancies, there are therapeutic agents that preferentially disrupt other adhesion interactions known to exist in the BM, including those mediated by CD44 and very late antigen-4 (VLA-4; integrin α4β1) (102).

BM niches are also a favorable destination for metastasizing cancer cells. Utilizing the same logic above, the interruption of cellular mechanisms that misguide the ‘homing’ of malignant cells from the bloodstream into the BM stroma could be exploited as a targeted strategy to prevent or treat metastases (106). In preclinical animal models of metastatic prostate and breast cancer, accumulating evidence supports the roles of CXCR4 and E-selectin in driving cancer cell dissemination in the BM, where they form metastases. Blockage of these target molecules leads to cancer cell mobilization and chemosensitization (107–109). The cancer-stroma interactions mediated by CXCL12/CXCR4 and E-selectin, which facilitate the formation of metastases in other anatomical sites, such as lymph nodes and lungs, are discussed elsewhere (16, 102).

Glioblastoma and a variety of other intracranial tumors are able to trigger the sequestration of T cells in the BM, resulting in T-cell lymphopenia and systemic tumor-promoting immunosuppression. The mechanism for this appears to be the loss of the sphingosine-1-phosphate receptor 1 on the T cell surface (110). This observation suggests that tumors have the ability to employ BM niches as another mechanism of immune escape; it is worth further exploring if this generally applies to non-cranial cancer entities. Counteracting this effect could enhance many immuno-oncological therapeutic strategies.

**BOX 1 Novel modulators of the TME: L-RNA aptamers as chemokine-neutralizing compounds.**

Therapeutic neutralization of secreted ligands confers an advantage over the blockage of their receptors because it has no direct action on cells, some of which belong to normal tissues. Endogenous ligand neutralization is typically accomplished by the biotechnological development of specific mAbs or ‘ligand traps’ (i.e., recombinant ligand-binding receptor domains). In contrast to protein-based biologics, L-RNA aptamers are immunologically inert. These synthetic compounds are made of nuclease-resistant PEGylated L-stereoisomer oligonucleotides that specifically bind ligands in a manner similar to antibodies. Two chemokine-neutralizing L-RNA aptamer drugs that modulate the TME are currently in development for oncology indications: (1) the CXCL12-neutralizing NOX-A12 (olaptesed pegol); (2) the CCL2-neutralizing NOX-E36 (emapticap pegol).

NOX-A12 in Glioblastoma

In combination with radiotherapy, antagonists to CXCL12, CXCR4 and CXCR7 have led to complete brain tumor regression and survival prolongation of tumor-bearing rodents (82, 111–113). Radiotherapy, the backbone of the standard of care for gliomas, has the unintended effect of promoting tumor recurrence *via* CXCR4/CXCR7-dependent vasculogenesis (discussed under ‘Angiogenesis and vasculogenesis inhibitors’). This undesirable pathological tumor reaction illustrates why anti-angiogenesis medication is insufficient as a salvage measure to curb post-radiotherapy tumor progression (17), clinically confirmed in glioblastoma patients (114). Additionally, endothelium-derived CXCL12 attracts MDSCs into the TME and contributes to its immunosuppressive characteristics (discussed under ‘Modulating the immune TME’), particularly in glioblastoma (115). Noteworthy, the CXCL12/CXCR4/CXCR7 axis is functionally involved in cancer cell proliferation, survival, and migration (16). Moreover, both individual CXCL12 receptors, i.e., CXCR4 and CXCR7, are implicated in brain tumor growth *in vitro* and *in vivo* (82, 111, 112). Thus, by blocking signaling *via* both CXCL12 receptors, NOX-A12 targets the whole axis, suggesting an advantage for NOX-A12 over individual CXCR4 and CXCR7 antagonists. In the absence of irradiation, the combination treatment with NOX-A12 and anti-VEGF mAb demonstrates a synergistic effect (116). In a phase 1/2 study of newly-diagnosed glioblastoma patients, plerixafor has been concurrently administered with chemoradiotherapy (83). In comparison to a contemporaneously assembled control group, locally diminished blood perfusion has been detected, supporting the hypothesized anti-vasculogenic effect of CXCR4 blockade. The safety and efficacy of NOX-A12 have been evaluated in combination with radiotherapy in newly-diagnosed glioblastoma patients characterized by the poor prognosis marker of unmethylated MGMT promoter (117). Interim results demonstrate a reassuring safety profile, a reduction in tumor size in 90% of the patients, and an objective response in 40%. This finding was accompanied by lower tumor cell proliferation, loss of CXCL12 expression in the endothelium, and the emergence of T-cell clusters. Together, these findings suggest re-shaping the TME to a less pro-tumoral microenvironment. Presently, the safety and potential synergy of NOX-A12 with radiotherapy and either VEGF inhibition or ICI are further explored in glioblastoma patients.

NOX-A12 in other solid tumors

A major hurdle for immunotherapies is that many tumors are, to some extent, ‘immune-excluded,’ characterized by an immunosuppressive TME and low numbers of infiltrating tumor-reactive lymphocytes. Thus, the efficacy of immunotherapies that stimulate immune cells, such as ICIs or cellular therapies, is inadequate if the immunosuppressive TME is not tackled simultaneously (discussed under ‘Enhancement of anti-tumor immune response’ and ‘Modulating the immune TME’) (3, 4). CXCL12/CXCR4 blockade is one of the mechanisms by which the immunosuppressive TME could be overcome, enhancing susceptibility to successive immunotherapy. This has been proven to be the case in animal models of various cancer types (86, 87, 118–122) as well as human spheroid/organotypic cultures (123, 124). Recently, this therapeutic approach has been employed in human trials to combat immunotherapy-resistant tumors, i.e., refractory microsatellite stable (MSS) PDAC and CRC. The delivery of CXCR4 antagonists or the CXCL12 antagonist NOX-A12 in those patients elicits Th1 immune response (62, 63, 125, 126), as detected by immunological assessment of paired biopsies. Following CXCL12 inhibition by NOX-A12, clusters of activated T cells have been observed in the treated CRC and PDAC patients (126), similar to findings in the glioblastoma patients (3, 117). These findings imply a reduced immunosuppressive pressure in the TME, allowing a more robust and coordinated anti-tumor immune response. Further development will comprise a clinical trial to determine the efficacy of NOX-A12 plus pembrolizumab therapy in second-line PDAC patients in the background of standard-of-care chemotherapy. Based on the suggested mechanism of an improved anti-cancer immune response, NOX-A12 has therapeutic potential in conjunction with ICIs for multiple oncological indications.

NOX-A12 in hematological malignancies

Administration of the CXCR4 antagonist plerixafor in combination with G-CSF is a well-established allogeneic/autologous HSC mobilization method. Mobilized HSCs are then collected from the PB by apheresis and engrafted in cancer patients with ablated BM to allow for the repopulation of the hematopoietic system. Mobilization/chemosensitization is an attractive strategy to mobilize and eliminate chemoresistant CXCR4^+^ malignant cells (94–99) (discussed under ‘Targeting the niche’). NOX-A12 inhibits CXCR4^+^ cell migration towards a CXCL12 gradient and is an efficient mobilizing agent in mice, non-human primates, and humans (127). Similarly, it can inhibit the migration and induce the mobilization of malignant cells, which rely on CXCL12/CXCR4 interactions to colonize the BM, as demonstrated in human CLL-stroma co-cultures (101) and murine models of MM and CML (128, 129). Interestingly, CXCL12 neutralization by NOX-A12 chemosensitizes CLL, MM, and CML cells resulting in their reduced survival *in vitro* or tumor burden *in vivo* (101, 128, 129). The clinical potency of NOX-A12 has been evaluated as a treatment for hematological malignancies. In phase 2a trials, R/R MM and CLL patients have been treated with escalating doses of NOX-A12 in combination with bortezomib and dexamethasone or bendamustine and rituximab, respectively (100, 130). In corroboration with the preclinical models (101, 128), NOX-A12 treatment effectively mobilizes myeloma and leukemic cells, maintaining high levels in the blood circulation for at least 72 hours (100, 130). The observed overall response rates (e.g., 68% for MM and 86% for CLL) are comparable to similar combination treatments with other novel agents, such as BTK and PI3K inhibitors. However, larger controlled studies are required to accurately validate the efficacy of NOX-A12 as a strategic combination therapy.

NOX-E36

As a major attractant of BM-derived cells that contribute to the creation of a tumor-supportive TME, the CCL2/CCR2 axis is of clinical relevance (discussed under ‘Modulating the immune TME’) (15). Since TAM accumulation is observed in various solid tumors and pharmacological CCR2 inhibition has proven effective in several cancer models, NOX-E36 provides translational potential. Preclinical and early clinical studies of advanced PDAC, in which CCR2 antagonists have been assessed with or without standard chemotherapy, show encouraging results epitomized by CCR2^+^ myeloid cell depletion (43–47). Corroborating these results, reduced TAMs are observed in murine PDAC and HCC models following treatment with a rodent version of NOX-E36 (mNOX-E36) (131, 132). Interestingly, mNOX-E36 also suppresses pathogenic angiogenesis and tumor blood flow in rodent models of glioblastoma and fibrotic HCC, supporting the pro-angiogenic function of TAMs (132, 133). In addition to antagonizing CCL2, NOX-E36 also antagonizes the closely related chemokines CCL8, CCL11, and CCL13 (134). Thus, NOX-E36 inhibits the activity of multiple monocyte/macrophage-relevant components of the innate PD-1 resistance (IPRES) signature (135). IPRES components are known to be implicated in creating an immune-privileged TME *via* the recruitment of immunosuppressive cells. NOX-E36 has already been shown to be safe in humans, and a clear pharmacodynamic effect has been established, i.e., the reduction of peripheral monocyte counts and their respective surface CCR2 expression. These observations confirm that NOX-E36 modulates a potentially immunosuppressive cell population in the TME. Clinical development of NOX-E36 for solid tumors is in planning.

## Future perspectives

This review lays out current therapeutic strategies to combat cancer progression by targeting the TME rather than malignant cells directly. The combination of this approach with established (e.g., chemoradiotherapy) or newer treatment strategies (e.g., targeted molecular therapy) holds great promise to overcome the limitations and challenges of the clearly insufficient current standard of care in many oncology indications. In the majority of patients treated with standard of care, including chemotherapy regimens and targeted molecular therapies, the combined effects of treatment resistance and selective pressure under treatment result in tumor progression. This is also the case for immunotherapies (e.g., anti-cancer antigen mAbs and ICIs) that rely on harnessing the immune system to eliminate cancer cells. Real-world evidence shows that a relatively small subset of cancer entities are highly susceptible to immunotherapy, and only a fraction of these patients exhibit a lasting clinical benefit in the case of approved treatments (3, 4, 27). Several escape pathways are implicated following immunotherapy, such as cancer antigen escape (e.g., downregulation of CD19 or CD20), alterations in downstream signaling events (e.g., induction of alternative survival pathways or resistance to antibody-dependent cellular cytotoxicity), and T-cell exhaustion (136). Lack of unique cancer antigens or loss of MHC class I and β2-microglobulin expression, which are responsible for antigen presentation on tumor cells, are known underlying mechanisms for the failure of anti-PD-1/PDL-1 therapy (3, 4). As discussed above, the abundance of immunosuppressive cytokines and cells in the TME and the exclusion of tumoricidal immune cells from the TME are key contributing factors to the development of immunotherapy resistance, whether intrinsic (existed pre-treatment) or acquired (arises post-treatment).

In contrast to the malignant cells of the tumor (1), the cells of the TME are non-cancerous, therefore the principles of clonal heterogeneity and evolution do not play a role. Moreover, the cellular TME components are not characterized by dynamic cell-autonomous immune escape and drug resistance mechanisms. Furthermore, non-cellular TME components, such as the modified ECM and various bioactive paracrine molecules, may indirectly contribute to the development of drug resistance in cancer cells. Detailed understanding of TME biology and the interplay between its components (e.g., stromal rearrangements, biomarker panels, the tumor microvasculature, secreted cytokines and chemokines, and the inflammatory immune cell milieu, all of which can be analyzed by comprehensive histopathological and genetic tools) at different stages of cancer progression is therefore important in order to design therapies with greater benefit for patients. Notably, once specific resistance mechanisms are characterized, they can be addressed by targeting the respective TME component. Clinical efforts aimed at targeting TME-localized immunosuppressive myeloid cells are one recent example of novel approaches to address the tumor-supportive TME. The hypothesis behind these trials is that elimination or reprogramming of immunosuppressive TME components could be an effective immunotherapeutic strategy in itself or in combination with the stimulation of effector immune cells. Beyond anti-tumor immunity, the contribution of tumor vascularization and supporting stroma to tumor progression and recurrence should be considered in the design of novel TME-targeting agents. TME-focused research is expected to identify new TME components with the potential for precise targeting, taking into account characteristics such as tumor sub-type and previously received anti-cancer treatments. Precision oncology on the basis of TME features is a promising therapeutic avenue that could massively improve the therapeutic benefit for patients with aggressive cancers. In conclusion, a thorough understanding of the TME and novel combination therapies which target multiple TME components in addition to conventional anti-cancer drugs have great potential to address the unmet medical need in oncology.

## Author contributions

DE contributed substantially to the conception and scope of the review and revised critically for important intellectual content; AF provided important sections and revised the review critically for important intellectual content; KL drafted the review; AM contributed substantially to the conception and scope of the review and revised critically for important intellectual content; AB provided important sections and revised the review critically for intellectual content. All authors contributed to the article and approved the submitted version.
