# Supplementary material for: The prospect of tumor microenvironment-modulating therapeutical strategies
Source: Front Oncol. 2022 Dec 8;12:1070243. doi: 10.3389/fonc.2022.1070243 (PMC9772844; doi:10.3389/fonc.2022.1070243)
Supplement: Supplementary file 1 [file Table_1.docx]

**Supplementary Table 1**

**TME-targeting combination therapies – ongoing clinical studies of interest**

| **Section in the main text** | **Investigational medicinal agents** | **Target molecules;** combination therapy targets | **Cancer type** | **Highest phases in development** | **Combination therapy** | **Clinical trial number** |
| --- | --- | --- | --- | --- | --- | --- |
| **Immuno-stimulation** | Bintrafusp alfa (M7824) | **TGFβ and**  **PD-L1** | NSCLC | 3 |  | NCT05061823 |
|  |  |  | NSCLC^1^, STS^1^, GGEEC^1^ | 2 | (+chemotherapy -/+radiotherapy) | NCT04396535  NCT04874311 NCT04595149  NCT04481256  NCT03840902 |
|  | SHR-1701 |  | GGEEC^1^ | 3 | (+chemotherapy) | NCT04950322 |
|  | Bintrafusp alfa (M7824) + NHS-IL12 (M9241) | **TGFβ and**  **PD-L1**; **IL-12R** | HPV-associated Cancers^1,2^, MSS CRC^1^, Prostate Cancer^1^, Kaposi Sarcoma^1^ | 1/2 | (-/+chemotherapy) | NCT04708470  NCT04633252  NCT04303117 |
|  | VG161 | **IL-12R, IL-15R and PD-L1** | HCC^1^ | 2 |  | NCT05223816 |
|  |  |  | Pancreatic Cancer^1^ | 1/2 | (+nivolumab) | NCT05162118 |
|  | Bempegaldesleukin (NKTR-214) | **IL-2Rβγ**;  PD-1; also targeted therapy | HNSCC^1^ | 2 | (+radiotherapy +pembrolizumab) | NCT04936841 |
|  |  |  | STS^1^ | 2 | (+nivolumab) | NCT03282344 |
|  | Nemvaleukin alfa (ALKS 4230) |  | Melanoma^1,2^, HNSCC, OFTPC^2^ | 2, 3 | (-/+pembrolizumab, -/+chemotherapy) | NCT04830124  NCT04144517  NCT05092360 |
|  | SAR444245  (THOR-707) |  | Pleural Mesothelioma, NSCLC, B Cell Lymphoma^2^ | 2 | (-/+pembrolizumab, -/+chemotherapy) | NCT04914897  NCT05179603 |
|  |  |  | Gastrointestinal Cancers^1^, HNSCC | 2 | (-/+pembrolizumab, -/+cetuximab) | NCT05104567  NCT05061420 |
|  | ALT-803  (N-803, Anktiva) | **IL-2R and IL-15R**; adjuvant or PD-1 | OFTPC^1^ | 2 |  | NCT03054909 |
|  |  |  | NSCLC^1^ | 1/2 | (+nivolumab) | NCT02523469 |
|  |  |  |  | 3 | (-/+chemotherapy +pembrolizumab) | NCT03520686 |
|  |  |  | Pancreatic Cancer^1^ | 2 | (-/+chemoradiotherapy) | NCT04390399 |
|  |  |  | Bladder Cancer (high grade non-muscle invasive) | 2/3 | (+adjuvant) | NCT03022825 |
|  | NT-I7  (GX-I7, TJ107, Efineptakin alfa) | **IL-7R** | Glioblastoma^1^ | 2 | (+chemoradiotherapy) | NCT04600817 |
|  |  | **IL-7R**;  PD-1 or PD-L1 | Breast Cancer (triple negative)^1^, HNSCC^1^ | 2 | (-/+chemotherapy +pembrolizumab) | NCT05145907 |
|  |  |  | NSCLC^1^ | 2 | (+atezolizumab) | NCT04984811 |
|  |  |  | GGEEC^1^ | 2 | (+nivolumab) | NCT04594811 |
|  | Magrolimab  (Hu5F9-G4) | **CD47**;  also targeted therapy | Myelodysplastic Syndrome | 3 | (+chemotherapy) | NCT04313881 |
|  |  |  | AML, AML (TP53 mutant) | 3 | (+chemotherapy -/+venetoclax) | NCT05079230  NCT04778397 |
|  |  |  | B Cell Lymphoma^2^ | 1 | (+venetoclax +obinutuzumab) | NCT04599634 |
|  |  |  | Non-Hodgkin Lymphoma^2^ | 1/2 | (-/+chemotherapy +rituximab) | NCT02953509 |
|  |  |  | MM^2^ | 2 | (-/+daratumumab -/+ proteosome inhibitors) | NCT04892446 |
|  |  |  | Breast Cancer (triple negative)^1^ | 2 | (-/+chemotherapy -/+sacituzumab govitecan) | NCT04958785 |
|  |  | **CD47**;  PD-1 | Hodgkin Lymphoma^2^ | 2 | (+pembrolizumab) | NCT04788043 |
|  |  |  | HNSCC | 2 | (-/+pembrolizumab -/+zimberelimab) | NCT04854499 |
|  | Evorpacept (ALX148) | **CD47**;  also targeted therapy | Myelodysplastic Syndrome | 1/2 | (+chemotherapy) | NCT04417517 |
|  |  |  | B Cell Lymphoma^2^ | 1/2 | (+rituximab +lenalidomide) | NCT05025800 |
|  |  |  | AML | 1/2 | (+chemotherapy -/+venetoclax) | NCT04755244 |
|  |  |  | HER2^+^ Cancers | 1/2 | (+zanidatamab) | NCT05027139 |
|  |  | **CD47**;  PD-1; also targeted therapy | GGEEC^1^ (HER2^+^) | 2/3 | (+chemotherapy +ramucirumab  -/+trastuzumab) | NCT05002127 |
|  |  |  | HNSCC^1^ | 2 | (-/+chemotherapy +pembrolizumab) | NCT04675294  NCT04675333 |
|  |  |  | MSS CRC^1^ | 2 | (+cetuximab +pembrolizumab) | NCT05167409 |
| **Immuno- modulation** | Cabiralizumab (BMS-986227)  (-/+ BMS-986253) | **CSF-1R**;  PD-1, CD40,  or IL-8 | T Cell Lymphoma^2^, Pancreatic Cancer^1^ | 2 | (-/+chemotherapy +nivolumab) | NCT03927105  NCT03697564 NCT03336216 |
|  |  |  | Melanoma^1^, NSCLC^1^, RCC^1^ | 1 | (+sotigalimab -/+nivolumab) | NCT03502330 |
|  |  |  | HNSCC, HCC^1^ | 2 | (+nivolumab) (vs. BMS-986253) | NCT04848116  NCT04050462 |
|  | SNDX-6352 |  | BTC | 2 | (-/+chemoradiotherapy +durvalumab) | NCT04301778 |
|  | BMS-986253  (HuMax-IL8) | **IL-8**;  PD-1 | Myelodysplastic Syndrome^2^ | 1/2 | (-/+chemotherapy) | NCT05148234 |
|  |  |  | Prostate Cancer (hormone-sensitive) | 1/2 | (+nivolumab +hormone therapy) | NCT03689699 |
|  | BMS-813160  (-/+ BMS-986253) | **CCR2/5**;  PD-1 or LAG-3 or CTLA-4; IL-8 | Pancreatic Cancer and CRC^1^, Pancreatic Cancer^1^ | 1/2 | (+chemotherapy -/+nivolumab) | NCT03184870  NCT03496662 |
|  |  |  | NSCLC^1^, HCC^1^, RCC^1^ | 2 | (+nivolumab) (vs. BMS-986253)  (-/+relatlimab -/+ipilimumab) | NCT04123379  NCT02996110 |
|  | FLX475 | **CCR4**;  PD-1 or CTLA-4 | Gastric Cancer^1^ | 2 | (-/+pembrolizumab) | NCT04768686 |
|  |  |  | Melanoma^1^ | 2 | (+ipilimumab) | NCT04894994 |
|  | Reparixin | **CXCR1/2** | Breast Cancer^1^ | 2 | (+chemotherapy) | NCT05212701 |
|  | Plerixafor (AMD3100) | **CXCR4**;  PD-1 or PD-L1; TIGIT/VEGFR2; also targeted therapy | Pancreatic Cancer^1^ | 2 | (+cemiplimab) | NCT04177810 |
|  | Motixafortide  (BL-8040) |  |  | 2 | (+pembrolizumab/cemiplimab  -/+chemotherapy) | NCT02826486  NCT04543071 |
|  |  |  | GGEEC^1^ | 1/2 | (+chemotherapy -/+atezolizumab vs. tiragolumab/ramucirumab or other agents) | NCT03281369 |
|  | Anakinra (Kineret) | **IL-1R** | Pancreatic Cancer | 2 | (+chemotherapy) | NCT04926467 |
|  | NOX-A12 | **CXCL12**;  PD-1 | Pancreatic Cancer^1^ | 2 | (+pembrolizumab +chemotherapy) | NCT04901741 |
|  | Canakinumab | **IL-1β**;  PD-1; also targeted therapy | Melanoma^1^ | 2 | (+spartalizumab) (vs. other agents) | NCT03484923 |
|  |  |  | Myelodysplastic Syndrome, CML | 2 | (+chemotherapy) | NCT04239157 |
|  |  |  | NSCLC^1^ | 2 | (-/+pembrolizumab) | NCT03968419 |
|  |  |  |  | 1/2 | (+durvalumab +chemoradiotherapy) | NCT04905316 |
|  | Certolizumab | **TNFα**;  PD-1 or CTLA-4 | Lung Cancer | 2 | (+chemotherapy) | NCT04991025 |
|  | Infliximab |  | Melanoma^1^ | 2 | (+pembrolizumab) | NCT05034536 |
|  | Tocilizumab | **IL-6R**;  also targeted therapy | Pancreatic Cancer^1^, Esophageal Cancer | 2 | (+chemotherapy -/+ radiotherapy) | NCT02767557  NCT04554771 |
|  |  | **IL-6R**;  PD-1 or PD-L1 or CTLA-4; A2aR/A2bR or TIGIT | Diffuse Astrocytoma (IDH-wildtype) or Glioblastoma^1^ | 2 | (+atezolizumab +radiotherapy) | NCT04729959 |
|  |  |  | Prostate Cancer, HNSCC^1^ | 2 | (+atezolizumab) (vs. etrumadenant/ tiragolumab) | NCT03821246  NCT03708224 |
|  |  |  | Melanoma^1^, NSCLC^1^, Urothelial Cancer^1^ | 2 | (+nivolumab +ipilimumab) | NCT04940299  NCT03999749 |
|  | Talimogene Laherparepvec (TVEC) | **GM-CSF**;  PD-1 | STS^1^ | 2 | (+nivolumab +chemotherapy) | NCT03886311 |
|  |  |  | Melanoma^1,2^, STS^1^, Skin Cancers^1,2^, Lymphoma^1^ | 2 | (-/+pembrolizumab/nivolumab) | NCT02965716  NCT04068181  NCT03069378  NCT03842943  NCT04330430  NCT02978625 |
| **Anti-angiogenesis/**  **vasculogenesis**  **+** **Immuno- stimulation or Immuno-**  **modulation** | Bevacizumab (or biosimilar) + ICIs | **VEGF-A**;  PD-1 or PD-L1 or CTLA-4; also targeted therapy | HCC, HCC^1^, Cervical Cancer^1^, OFTPC^1^, Pleural Mesothelioma^1^, Breast Cancer (HR-positive HER2-negative)^1^, CRC (dMMR or MSI high)^1^, NSCLC^1^ | 3 | (-/+atezolizumab -/+chemotherapy)  (-/+TKIs or other agents) | NCT03556839  NCT04487067  NCT04102098  NCT03038100  NCT03762018  NCT04732598  NCT02997228  NCT03353831  NCT02891824  NCT03991403  NCT04194203  NCT03434379  NCT04803994  NCT04712643 |
|  |  |  | OFTPC^2^, Cervical Cancer^1^, OFTPC (BRCA non-mutated)^1^, Cervical Cancer^1^, CRC^1^ | 3 | (-/+pembrolizumab -/+chemotherapy)  (-/+cetuximab, olaparib or other agents) | NCT05116189  NCT03635567  NCT03740165  NCT05239741 |
|  |  |  | OFTPC^1^, HCC, HCC^1^ | 3 | (-/+durvalumab +chemotherapy)  (-/+olaparib or other agents) | NCT03737643  NCT03778957  NCT03847428 |
|  |  |  | CRC (dMMR or MSI-H)^1^ | 3 | (-/+nivolumab -/+ipilimumab  -/+chemotherapy) (-/+cetuximab) | NCT04008030 |
|  |  |  | Cervical Cancer | 3 | (+chemotherapy -/+AK104) | NCT04982237 |
|  |  |  |  | 3 | (+chemotherapy -/+BCD-100) | NCT03912415 |
|  |  |  | NSCLC^1^ | 3 | (+chemotherapy -/+HLX10) | NCT03952403 |
|  |  |  | Ovarian Cancer^1^, OFTPC^1^ | 3 | (+chemotherapy -/+dostarlimab)  (-/+niraparib or other agents) | NCT03602859  NCT04679064 |
|  |  |  | HCC^1^ | 3 | (+chemotherapy -/+toripalimab) | NCT04723004 |
|  |  |  | CRC (RAS mutant), HCC^1^, NSCLC (EGFR mutant)^1,2^ | 3 | (-/+chemotherapy -/+sintilimab) | NCT04194359  NCT04682210  NCT03802240 |
|  | Bevacizumab (or biosimilar) + other immunostimulant/  modulant agents | **VEGF-A**;  TGFβ or IL-7R or CSF-1R | Cervical Cancer^1^, NSCLC (EGFR mutant)^1,2^ | 3 | (+chemotherapy -/+SHR-1701) | NCT05179239  NCT05132413 |
|  |  |  | Glioblastoma^1^ | 2 | (+GX-I7) | NCT05191784 |
|  |  |  | OFTPC^2^ | 2 | (+chemotherapy -/+emactuzumab) | NCT02923739 |
|  | Ramucirumab + ICIs | **VEGFR2**;  PD-1 or PD-L1 | GGEEC^1^, NSCLC, NSCLC^1^, NSCLC (EGFR mutant)^1^ | 2 | (-/+chemotherapy -/+pembrolizumab) | NCT04632459  NCT04040361  NCT03971474  NCT04120454  NCT04069273 |
|  |  |  | NSCLC^1^, Mesothelioma | 2 | (-/+chemotherapy -/+nivolumab -/+TKIs) | NCT04310007  NCT03527108  NCT03502746 |
|  |  |  | NSCLC^1^ | 2 | (+atezolizumab) | NCT03689855 |
|  |  |  | GGEEC^1^ | 2/3 | (-/+chemotherapy +sintilimab) | NCT04675983 |
|  | NOX-A12 | **CXCL12**;  VEGF-A or PD-1 | Glioblastoma (MGMT-unmethylated)^1^ | 1/2 | (+radiotherapy) (-/+bevacizumab  -/+pembrolizumab) | NCT04121455 |
|  | Plerixafor (AMD3100) | **CXCR4** | Glioblastoma^1^ | 2 | (+chemoradiotherapy) | NCT03746080 |
| **Stroma/Niche** |  |  | AML, ALL | 2 | (+chemotherapy) | NCT02605460 |
|  | Motixafortide  (BL-8040) |  | AML, T Cell Lymphoma^2^ | 2 | (+chemotherapy) | NCT02502968  NCT02763384 |
|  | Uproleselan  (GMI-1271) | **E-Selectin** | AML^2^ | 3 | (+chemotherapy) | NCT03616470  NCT05054543 |
| **Stroma +**  **Immuno-stimulation** | Darleukin, Bifikafusp alfa  (L19-IL2) | **Fibronectin extra domain B**; **IL-2R, IL-12R or TNFRSF**;  also targeted therapy | Diffuse large B cell lymphoma^2^ | 1/2 | (+rituximab) | NCT02957019 |
|  |  |  | NSCLC^1^ | 2 | (+radiotherapy) | NCT03705403 |
|  | Fibromun, Onfekafusp alfa  (L19-TNF) |  | STS^1^ | 3 | (+chemotherapy) | NCT04650984 |
|  | Daromun  (L19-IL2 + L19-TNF) |  | Melanoma^1^ | 3 | (-/+adjuvants) | NCT03567889 NCT02938299 |

For each combination therapy, the highest phase trials in development are indicated. Earlier studies are indicated if mentioned in the main text. Up-to-date as of 21.07.2022.

^1^ Advanced, recurrent, metastatic or high risk; ^2^ Relapsed/refractory. Chemotherapeutical agents and the type of radiotherapy are not indicated; **In bold** – the primary target molecule of the investigational medicinal agent; not in bold – combination targets; ‘also targeted therapy’ – drugs against a specific oncogene, e.g., the EGFR inhibitor cetuximab.

Abbreviations: ALL, Acute Lymphocytic Leukemia; AML, Acute Myeloid Leukemia; BTC, Biliary Tract Cancer; CML, Chronic Myeloid Leukemia; CRC, Colorectal Cancer; dMMR, deficient DNA Mismatch Repair; EGFR, Epidermal Growth Factor Receptor; GGEEC, Gastric or Gastro-esophageal Junction or Esophageal Cancers; HCC, Hepatocellular Cancer; HNSCC, Head and Neck Squamous Cell Cancer; HPV, Human Papillomavirus; HR, Hormone (estrogen and progesterone) Receptor; MM, Multiple Myeloma; MSI/MSS, Microsatellite Instable/Stable; NSCLC, Non-Small Cell Lung Cancer; RCC, Renal Cell Cancer; OFTPC, Ovarian, Fallopian Tube and Primary Peritoneal Cancers; STS, Soft-Tissue Sarcomas.
